# Corrosion of Sulfate-Reducing Bacteria on L245 Steel under Different Carbon Source Conditions

**DOI:** 10.3390/microorganisms12091826

**Published:** 2024-09-03

**Authors:** Ming Sun, Xinhua Wang, Wei Cui

**Affiliations:** 1 College of Mechanical & Energy Engineering, Beijing University of Technology, Beijing 100124, China; sm8014708@163.com; 2 China Special Equipment Inspection and Research Institute, Beijing 100029, China; 3 Technology Innovation Center of Oil and Gas Pipeline and Storage Equipment Safety, State Administration for Market Regulation, Beijing 100029, China

**Keywords:** shale gas, carbon steel, microbiologically influenced corrosion (MIC), sulfate-reducing bacteria, carbon source starvation

## Abstract

Objective Sulfate-reducing bacteria (SRB) pose a threat to the safe operation of shale-gas-gathering pipelines. Therefore, it is essential to explore the role played by SRB in dedicated pipelines. Methods In this work, the corrosion behavior of SRB was investigated by organic carbon starvation immersion experiments combined with cell number monitoring, corrosion weight loss recordings, morphology and profile observations and electrochemical measurements. Results In experiments with sodium lactate content ranging from 0–3500 ppm, the corrosion rate and pitting depth were the highest at 350 ppm. Conclusions The results indicated that the reduction in carbon sources leads to bacterial starvation, which directly obtains electrons from metals and exacerbates corrosion. It is not appropriate to use the content of bacteria to determine the strength of bacterial corrosion.

## 1. Introduction

Microorganisms are commonly found in soil, fresh water, oil fields, and ocean environments. SRB, saprophytic bacteria (TGB), and iron-oxidizing bacteria (IOB) are the main microbial groups that cause the local corrosion of gathering and transportation pipelines in oil and gas fields. Among them, SRB are the most widely reported microbial group and have the most harmful impact on pipelines because they may cause pitting or cracking of pipelines [1,2,3]. In recent years, with the development of shale gas, the cases of SRB corrosion caused by fracturing technology have increased significantly. The joint action of SRB corrosion with CO_2_ corrosion, Cl^−^ corrosion, and oil–gas erosion can greatly promote the corrosion and perforation of pipelines [4,5,6,7,8].

Traditionally, there are mainly four mechanisms of microbial pitting: (1) cathode depolarization theory, (2) concentration cell theory, (3) biocatalytic cathode reduction theory, and (4) submembrane acid corrosion theory. Recently, with the development of biotechnology and characterization methods, more and more research has been conducted on MIC. Some studies consider that SRB has two main corrosion types, namely metabolite MIC (M-MIC) and extracellular electron transfer MIC (EET-MIC). Among them, the M-MIC mechanism is the process of chemical corrosion between secreted metabolites as corrosive media and metal substrates, which are corrosive oxidants such as organic acids. The M-MIC pathway often leads to comprehensive corrosion of metals rather than localized pitting; for example, the metabolic products of SRB cause the copper substrate to become thinner as a whole without obvious local corrosion characteristics [9]. 

The EET-MIC refers to the fact that the SRB biofilm takes elemental iron or other high-energy metals as electron donors in the absence of carbon sources and other electron donors (which is necessary for microbial energy metabolism) and gains cathode electrons through extracellular electron transfer for energy generation. Since EET-MIC makes the bacteria more corrosive, this corrosion type has attracted additional attention from scholars in recent years [10]. Chen et al. [11] have shown that in the absence of organic electron donors, the SRB *D. vulgaris* can survive for a long time, up to 55 days, by directly attaching to the surface of carbon steel or in the FeS shell formed on carbon steel. The FeS shell can transfer electrons from the surface of carbon steel to *D. vulgaris* cells embedded in the FeS shell to aid their long-term survival, which is a typical process of EET-MIC. Xu et al. [12] have found that starving SRB with preformed biofilm can obtain energy by the Fe^0^ oxidation mechanism instead of the organic carbon oxidation mechanism. When the amount of carbon source in the medium was reduced by 90% and 99%, the corrosion weight loss increased, and the width of the pits became larger. Dou et al.’s [13] research shows that the most severe corrosion in terms of weight loss and maximum pit depth occurred with 20% carbon source level. The extreme starvation condition with 0% carbon source level did not corrode the most because it suffered from too much loss of sessile cells. Under carbon source starvation, SRB sessile cells were forced to use elemental iron to substitute lactate as the electron donor for sulfate reduction. This required EET. This biocorrosion study demonstrated that the carbon source starvation experimental strategy and various corrosion study techniques are useful tools for investigating microbial EET.

To sum up, SRB corrosion is an important corrosion factor in shale gas gathering and transportation pipelines. The supply level of carbon source affects the metabolic paths of bacteria, which further affects the corrosion rate of materials. This study conducted SRB corrosion research using corrosion immersion tests, characterized by different concentrations of sodium lactate at different levels of carbon sources, especially studying the changes in electrochemical parameters and surface morphology of the metal surface films and the pitting characteristics of the metal substrate. It is expected that our work can provide a theoretical basis for the control of SRB corrosion.

## 2. Materials and Methods

### 2.1. Bacteria Extraction, Identification, and Content Testing

Production water was obtained from a shale-gas-gathering and transportation system and then inoculated into the culture medium to expand the culture of bacteria. The composition of the production water is shown in Table 1, and the composition of the culture medium is shown in Table 2. The inoculation and culture methods refer to the standard GB/T 14643.5-2009 [14,15]. The SRB were cultured in the culture medium and then isolated from the medium. The isolated SRB were identified by 16s rDNA technology. According to the strain identification results, the main strain in the cultured SRB is *desulphovibrio*, which is considered a kind of mesothermal bacteria. The bacteria have arc-shaped or rod-shaped cells that appear single or in pairs. Simple organic compounds such as lactate can be used as electron donors and carbon sources for this kind of bacteria, and the bacteria belong to anaerobic bacteria with respiratory metabolism and fermentation metabolism functions [16,17].

Before and after the corrosion test, the planktonic cells in the solution and sessile cells on coupons were measured using SRB test vials purchased from Haitun (SRB-HX, Beijing, China), which is based on the MPN method. Three groups of parallel tests were conducted for each sample, and each group had 10 dilution concentrations. After inoculation, the test bottles were placed in a bacterial incubator at a constant temperature of 35 °C for one week. The color changes of the mixture in the test bottles were observed, and the bacterial content was calculated according to the standard.

### 2.2. Sample Preparation

**Figure 1:**
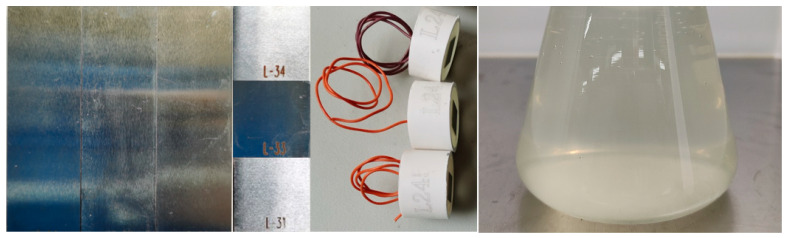
The samples and solution.

At present, L245 steel is mostly used for gathering and transportation pipelines in the bacterial-bearing blocks of the western Sichuan gas field. In this study, L245 steel pipes obtained on site were used to carry out the tests, and the composition of L245 steel is shown in Table 3. The test samples were cut in three different dimensions. Samples for the corrosion weight loss testing had dimensions of 60 mm × 20 mm × 3 mm. Samples for the microscopic morphology observation measured 20 mm × 20 mm × 3 mm. The dimensions of samples for the electrochemical testing were 10 mm × 10 mm × 3 mm. For the electrochemical samples, only the top surface was exposed to the solution, and the other surfaces were painted with Teflon coating. For other samples, all surfaces were exposed to the solution. Each group of samples with different dimensions comprised three samples. The surfaces of samples were pre-treated with a grinding machine and then polished with #800 sandpaper. After that, the samples were degreased with acetone, dried in cold air, and sterilized by ultraviolet light for 30 min on an ultra-clean workbench. The samples and solution are shown in Figure 1.

### 2.3. Corrosion Test

Before the corrosion test, the test container, polytetrafluoroethylene (PTFE) clamp, and deionized water were sterilized in a high-pressure steam sterilization pot at 121 °C for 15 min. The reagents were sterilized by ultraviolet light for 30 min on the ultra-clean workbench. The test solution was the simulated solution of production water in Table 1, and different contents of sodium lactate (0, 100 mg/L, 200 mg/L, 350 mg/L, 500 mg/L, 700 mg/L, 1750 mg/L, and 3500 mg/L) were, respectively, added to the simulated solution as the main carbon source. The experiment without inoculation with SRB under the condition of 350 mg/L sodium lactate was used as a blank control. The prepared test solution was deaerated with 99.99% nitrogen for 24 h. The test period was 10 days, the test temperature was 35 °C, while the test atmosphere was pure CO_2_. Before the corrosion test, each group was inoculated with the same amounts of bacteria.

### 2.4. Test and Characterization

After the corrosion test, the corrosion weight loss samples were taken out of the corrosive solution, washed with alcohol, and dried in cold air. Corrosion products were removed by a solution of 500 mL hydrochloric acid + 500 mL deionized water + 3.5 g hexamethylenetetramine refer to the requirements of GB/T 16545-2015 [18]. Then, the sample was weighed, and the corrosion rate was calculated. The samples for morphology observation were washed using alcohol and dried in cold air. The microscopic morphologies of the samples were observed using scanning electron microscopy (SEM, s-3400N, Hitachi, Japan) with the secondary electron (SE) mode and the surface elemental compositions were analyzed using energy dispersive spectrometry (EDS). The samples whose film layers were removed by acid washing were observed using SEM to study the pitting morphology and distribution of pits. The three-dimensional dimensions of the pits were measured by a three-dimensional stereo-microscope system.

Electrochemical impedance spectroscopy (EIS) with a three-electrode system and equipped with an electrochemical workstation (CHI 660E, CH Instruments, Shanghai, CN) were carried out. The reference electrode (RE) was a saturated calomel electrode (SCE), the counter electrode (CE) was a platinum electrode, and the working electrode (WE) was the L245 steel sample. The test parameters were as follows: the sine-wave excitation signal amplitude was 10 mV, the scanning frequency range was 10^−2^~10^5^ Hz, and the test temperature was 35 °C.

**Figure 2:**
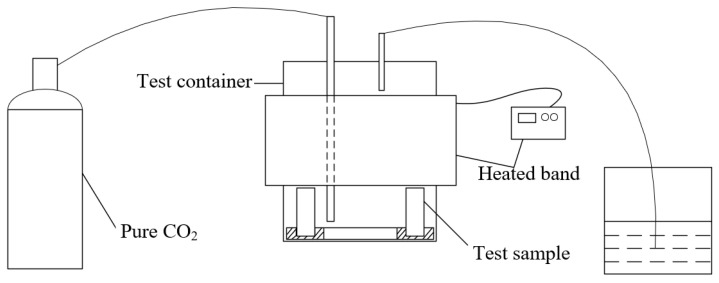
The schematic representation of experimental test system.

A schematic representation of the experimental test system is shown in Figure 2.

## 3. Results

### 3.1. Experimental Phenomenon and Bacteria Content Test Results

**Figure 3:**
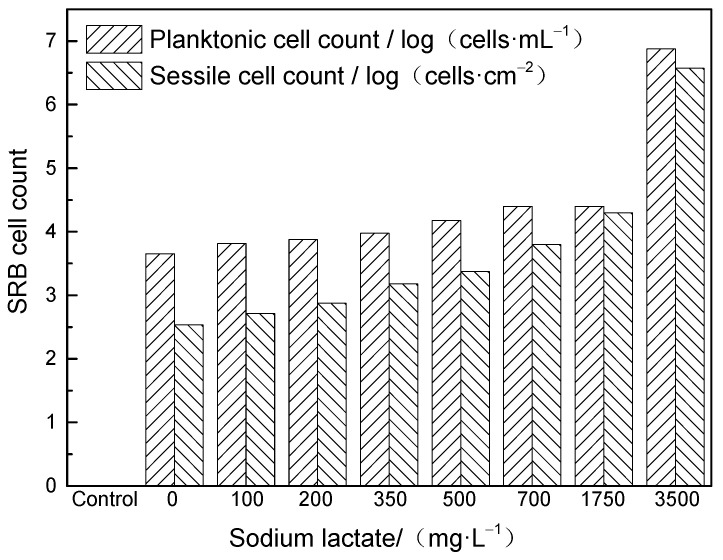
Variation of SRB concentration with sodium lactate content.

After the test, the state of the solution, solution odor, and sample conditions were recorded, as shown in Table 4. With the increase in carbon source content, the odor of rotten eggs became stronger, while the colors of solution and sediment became black. Figure 3 shows the variation of the planktonic cells in the solution and sessile cells on coupons with the increase in carbon source content. As can be seen, the initial concentration of bacteria was 450 cells/mL, and with the increase in carbon source content, the SRB content showed an increasing trend. When the sodium lactate content was 3500 mg/L, the bacteria content was two orders of magnitude higher than that under other conditions. The bacteria content was 0 in the control group.

**Figure 4:**
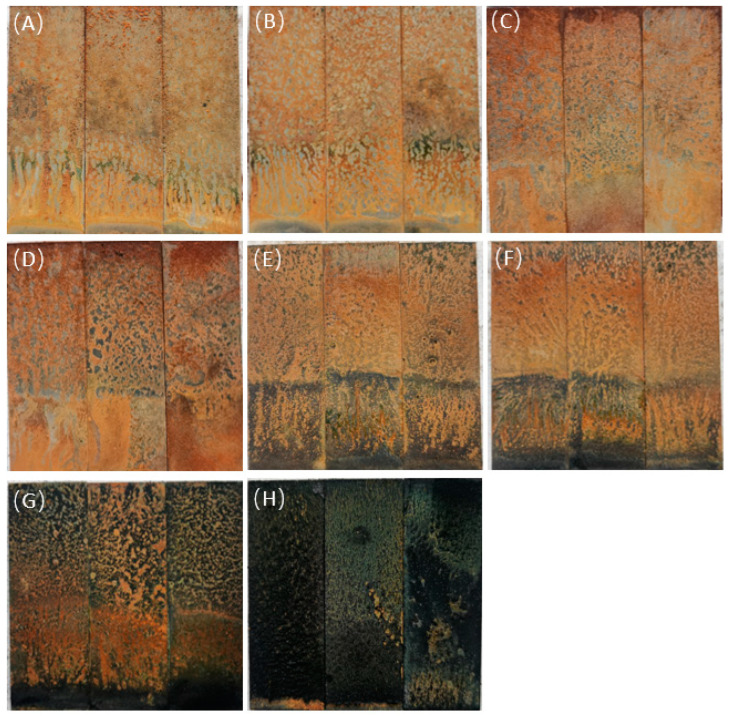
The macroscopic appearance of the sample surface with different content of sodium lactate. (**A**) 0; (**B**) 100 mg/L; (**C**) 200 mg/L; (**D**) 350 mg/L; (**E**) 500 mg/L; (**F**) 700 mg/L; (**G**) 1750 mg/L; (**H**) 3500 mg/L.

Observe the macroscopic morphology of the sample surface after the experiment, as shown in Figure 4. When the content of sodium lactate was 0~350 mg/L, the facial mask layer on the sample surface was mainly yellow; when the content of sodium lactate was 500~1750 mg/L, the facial mask layer on the sample surface was black and yellow, and the proportion of black gradually increased; when the content of sodium lactate was 3500 mg/L, the surface of the sample was a black film layer.

### 3.2. Corrosion Rate

**Figure 5:**
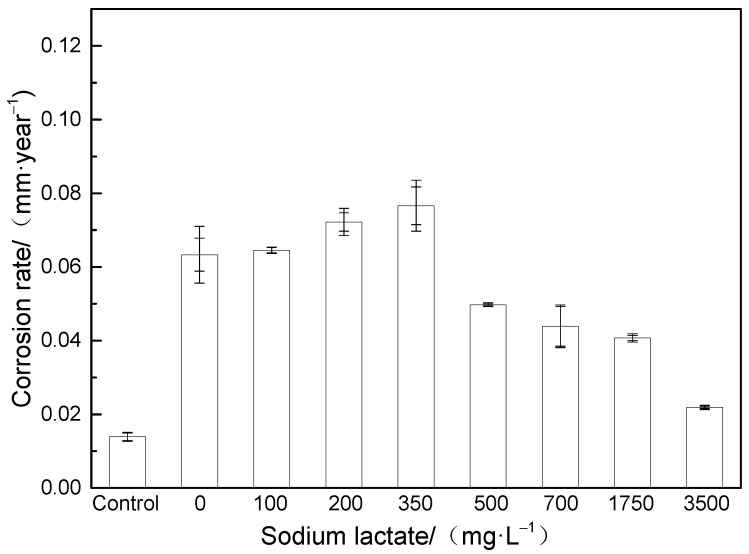
Changes in general corrosion rate of L245 carbon steel with content of sodium lactate.

The corrosion rate was calculated by the weight loss method, and the results are shown in Figure 5. When the sodium lactate content was 350 mg/L, the corrosion rate was the highest (0.077 mm/year). With the increase in sodium lactate content to 500–3500 mg/L, the corrosion rate gradually decreased to 0.022 mm/year. According to the test results of planktonic bacteria content, the bacterial content increased gradually with the increase in carbon source content, but the variation trend of corrosion rate was opposite to that of the bacterial content. 

When the content of sodium lactate was greater than or equal to 350 mg/L, as the content of sodium lactate increased, the content of planktonic bacteria increased and the corrosion rate decreased. The trend of corrosion rate change was opposite to that of bacterial content. At this time, there was sufficient organic electron donation, and bacteria obtained electrons from the organic electron donor. The corrosion of the metal substrate was inhibited, and the corrosion rate decreased. When the content of sodium lactate was less than or equal to 350 mg/L, as the content of sodium lactate decreased, the content of planktonic bacteria decreased and the corrosion rate decreased. The trend of corrosion rate change was the same as that of bacterial content. At this time, there were fewer organic electron donors, and bacteria sought metals as electron donors, which exacerbated corrosion. At the same time, when the content of sodium lactate was 0–200 mg/L, bacteria may have lacked the conditions for large-scale reproduction in the initial stage, the biofilm weakened, and necessary enzymes and other molecules were lacking, making it difficult to effectively attack the sample. However, when the content of sodium lactate was less than 350 mg/L, “hungry” bacteria still attacked the sample, and the corrosion rate was greater than when the content of sodium lactate was 500–3500 mg/L. This result is consistent with the research findings of Xu et al. [12]. The corrosion rate was lowest in the control group.

### 3.3. Microscopic Morphology Observation

**Figure 6:**
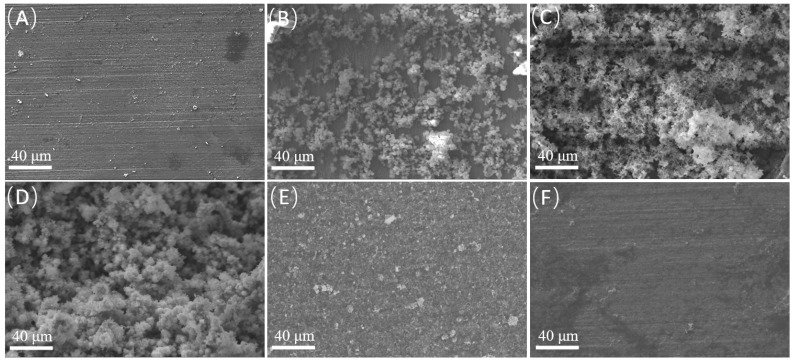
SEM observation images showing changes in microtopography with different content of sodium lactate: (**A**) control; (**B**) 0; (**C**) 350 mg/L; (**D**) 700 mg/L; (**E**) 1750 mg/L; (**F**) 3500 mg/L.

**Figure 7:**
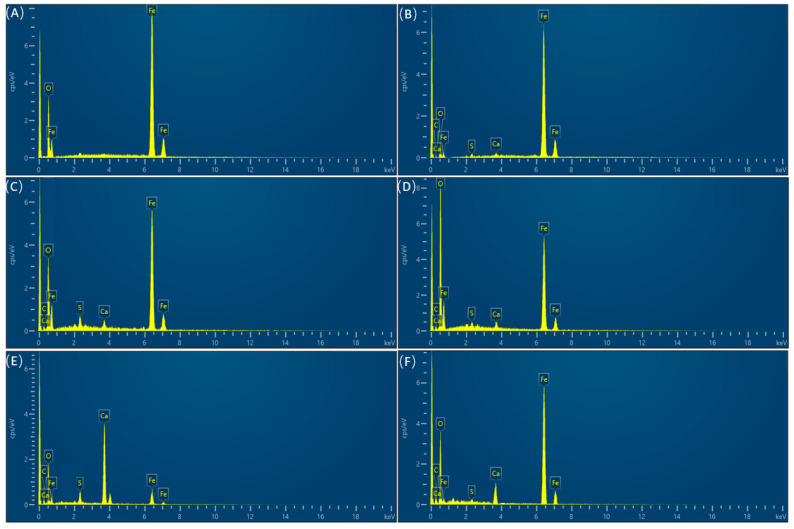
EDS spectrum analysis results derived from the sample surfaces (**A**) Control; (**B**) 0; (**C**) 350 mg/L; (**D**) 700 mg/L; (**E**) 1750 mg/L; (**F**) 3500 mg/L.

The samples before acid washing were observed using SEM, and EDS analysis was also conducted. Figure 6 and Figure 7 and Table 5 show the observation and analysis results. In the control group, the surface was flat, and scratches on the metal substrate were visible. There were no S, C and Ca in the control group; almost all elements were Fe. When the sodium lactate content was 0, a small amount of corrosion products and biofilms were distributed on the surfaces of the samples. When the content of sodium lactate was 350 mg/L, the white cluster film layer was clearly visible, and it was relatively loose. EDS spectra showed that there were C, O, S, Ca, and Fe elements in the film layer. Among them, the existence of the S element was important evidence that SRB participated in the corrosion process, while the high content of C and O elements indicated the existence of biological components. Hence, the surface products were mainly a mixture of SRB biofilm and corrosion product films formed after SRB participated in corrosion. When the content of sodium lactate was 700 mg/L, the film layer was denser and could protect the substrate to a certain extent. At the same time, the S content decreased to 1.83%, indicating that the degree of SRB participation in corrosion was reduced at this time. When the sodium lactate content was 1750 mg/L and 3500 mg/L, the film layer became denser, covering the entire substrate surface and providing protection to the substrate. At the same time, the S content decreased to 1.98% and 4.35%, respectively, indicating that the degree of SRB participation in corrosion was relatively low at this time. At a sodium lactate content of 350 mg/L, there were few organic electron donors, and the starving bacteria beneath the film turned to gain electrons from the surface of the metal substrate. Meanwhile, the loose film layer was conducive to the exchange of substances, which could speed up the corrosion process, resulting in the highest corrosion rate. Moreover, the largest S content indicated that more bacteria participated in the corrosion process. 

### 3.4. Pitting Corrosion Observation and Pitting Depth Measurement

**Figure 8:**
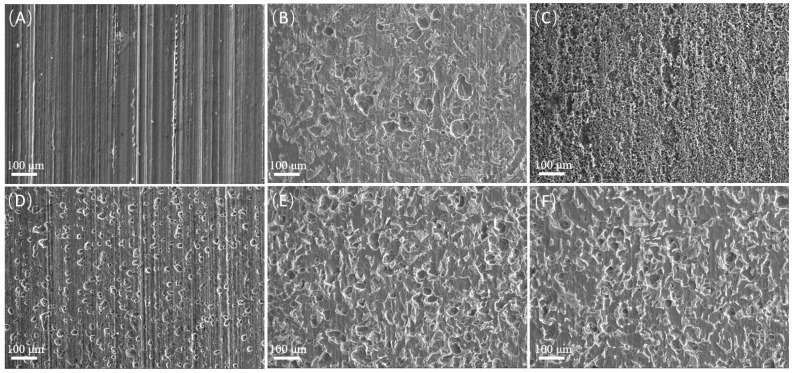
Microscopic observation of the samples after acid washing under SEM with different content of sodium lactate: (**A**) control; (**B**) 0; (**C**) 350 mg/L; (**D**) 700 mg/L; (**E**) 1750 mg/L; (**F**) 3500 mg/L.

The surface morphologies of the samples after acid washing were observed, and the results are shown in Figure 8. As can be seen, the pitting morphologies showed great differences at different carbon source contents. In the absence of sodium lactate, there were basically no corrosion pits. When the sodium lactate content was 350 mg/L, the corrosion pits were densely distributed, covering the whole surface of the substrate, and large corrosion pits were formed in some positions. When the sodium lactate content was 700 and 1750 mg/L, the corrosion pits were rounded and evenly distributed, and the number of corrosion pits was less than that when the sodium lactate content was 350 mg/L. When the sodium lactate content was 3500 mg/L, there were few corrosion pits. In the control group, the surface was scratched and there was no localized corrosion.

**Figure 9:**
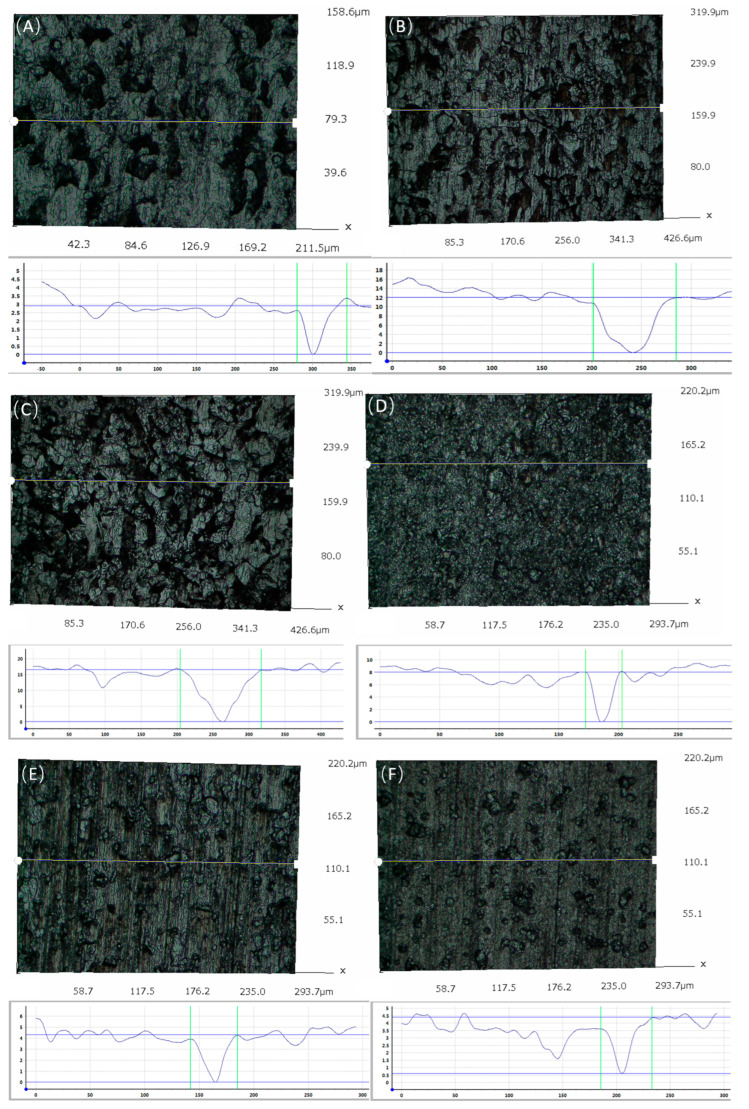
Pitting depths measured with a three-dimensional stereomicroscope system: (**A**) control; (**B**) 0; (**C**) 350 mg/L; (**D**) 700 mg/L; (**E**) 1750 mg/L; (**F**) 3500 mg/L.

**Figure 10:**
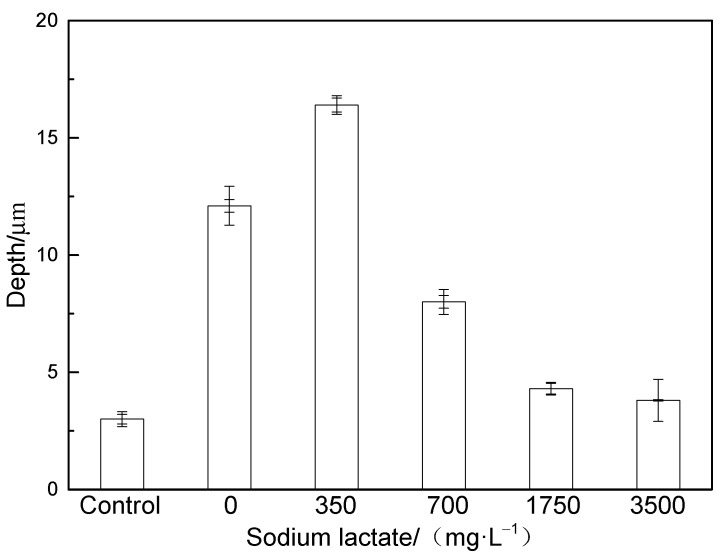
Pitting depth measured with a three-dimensional stereo-microscope system.

The typical pitting depths at different carbon source contents were measured using a three-dimensional stereomicroscope system. The typical pitting depths at different carbon source contents are shown in Figure 9 and Figure 10. The change trend of pitting depth was basically consistent with that of the corrosion rate, and the pitting depth reached its maximum value when the sodium lactate content was 350 mg/L, reflecting that both overall corrosion and local corrosion were prominent at this sodium lactate content. The depth of pitting was lowest in the control group.

When the content of sodium lactate was 0 and 350 mg/L, the carbon source was insufficient, and SRB bacteria directly used elemental iron as an electron donor, exhibiting the EET-MIC mechanism, strong corrosiveness, a large amount of pitting, and deep penetration; when the content of sodium lactate was 0, bacterial growth was inhibited. Although the bacteria were in a “hungry” state at this time, due to their small number and low activity, corrosion was somewhat inhibited. When the content of sodium lactate was greater than 350 mg/L, the carbon source was relatively sufficient, and bacteria had sufficient organic electron donors, exhibiting the M-MIC mechanism. With the increase in sodium lactate content, pitting corrosion decreased, and the depth was shallower.

### 3.5. Electrochemical Test

**Figure 11:**
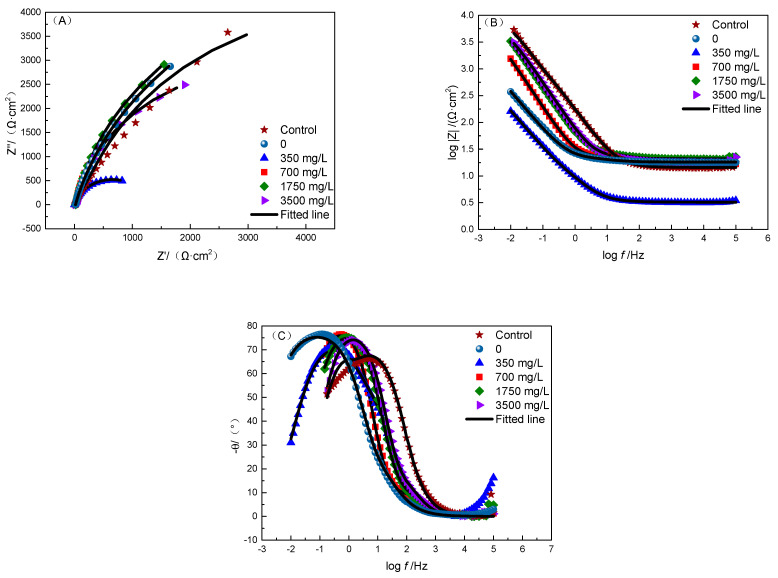
Electrochemical measurement results: (**A**) Nyquist plots of EIS results; (**B**,**C**) Bode plots of EIS results.

**Figure 12:**
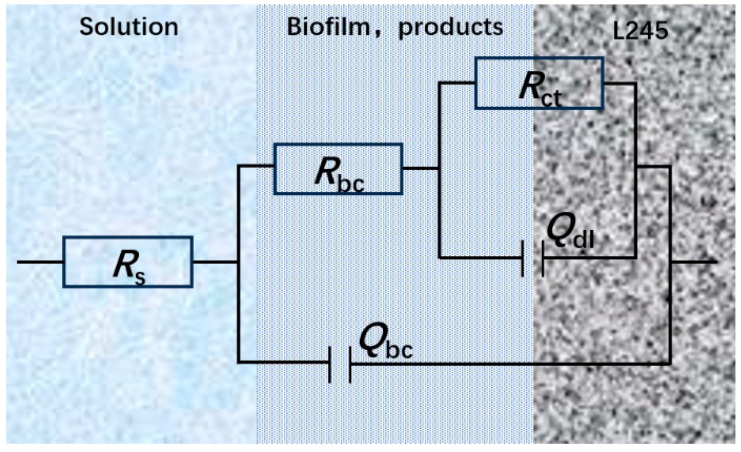
Equivalent circuits for EIS fitting.

With the change in solution proportioning, the biofilms and the product films on the sample surface also changed, which had some impacts on the electrochemical behavior. The electrochemical impedance spectra of the samples at different carbon source contents were tested, and the results are provided as follows. Figure 11 shows the Nyquest diagrams with fitting curves. In the EIS tests, a dual-time-constant circuit was used to simulate the equivalent circuit, as shown in Figure 12, and the fitting values are shown in Table 6. Here, *R*_s_, *R*_bc_, and *R*_ct_ represent the solution resistance, the resistance of biofilm and corrosion product layer, and the charge transfer resistance, respectively; *Q*_bc_ and *Q*_dl_ represent the capacitance of the biofilm and the corrosion product layer and the capacitance of the double electric layer, respectively. The key parameter *R*_bc_ + *R*_ct_ reflects the difficulty level of corrosion in the electrochemical circuit.

**Figure 13:**
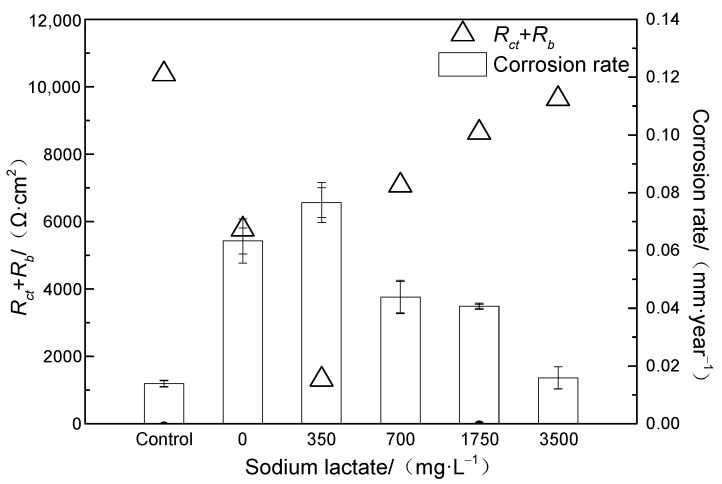
Curve of equivalent circuit parameters fitted by EIS with different contents of sodium lactate.

From the equivalent circuit parameters obtained by EIS fitting, it can be seen that *R*_s_ was basically stable at different sodium lactate contents. The increase in *R*_bc_ + *R*_ct_ value indicated that electron transfer was hindered, which led to a decrease in the corrosion rate. Figure 13 shows the relationship between the corrosion rates and *R*_bc_ + *R*_ct_ values. As can be seen, the corrosion rate was negatively correlated with the *R*_bc_ + *R*_ct_ value.

## 4. Discussion

**Figure 14:**
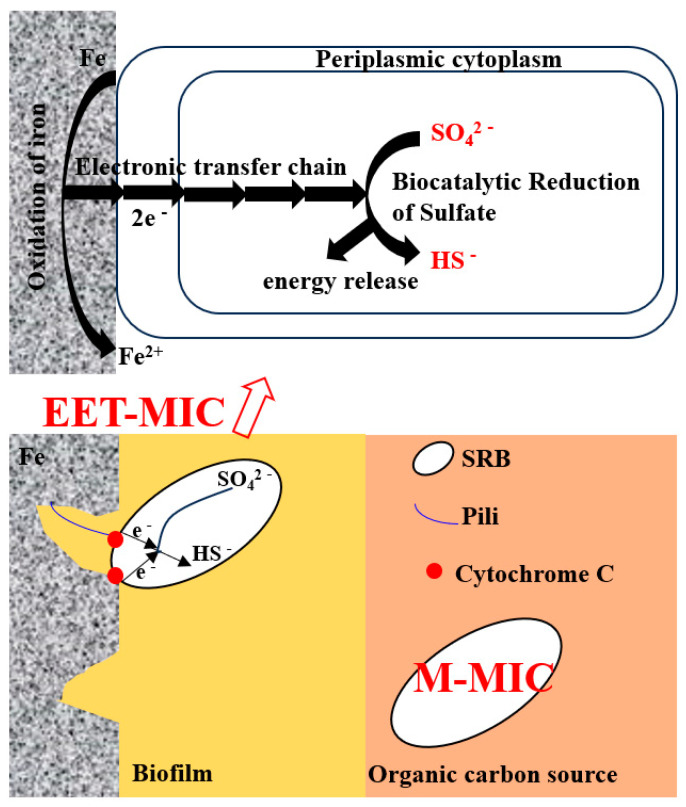
Bacterial corrosion mechanisms of EET-MIC and M-MIC.

Recent studies have shown that when mature SRB biofilms are starved of carbon sources, they will turn to elemental iron as an electron donor. Thus, the corrosion mechanism changes from M-MIC to EET-MIC, and the corrosivity of SRB becomes stronger. The research reported in the literature are compared in Table 7. The specific principles of SRB corrosion are illustrated in Figure 14. In this study, the sodium lactate content was used as the control variable to simulate bacterial corrosion under different carbon source supply conditions.

The results show that the effect of carbon source content change on bacterial growth and corrosion is a complex process. Under conditions of no or low carbon source content, such as in the experimental group without sodium lactate, the growth of bacteria was inhibited at the beginning stage, and there were few bacterial colonies on the surface of the metal substrate, without complete biofilms. Although the bacteria were in a “starvation” state, there were not enough bacteria to participate in the electron acquisition process from the metal substrate, so the growth of bacteria was inhibited and the corrosion was also hindered. In this case, the charge transfer resistance was high, and the corrosion rate and pitting depth were small. Therefore, the corrosion problem was not prominent. Under the condition of sufficient carbon source supply, such as in the experimental group with a sodium lactate content of 3500 mg/L, the bacteria had enough organic electron donors and did not face the “starvation” situation; moreover, a dense film could be formed to protect the substrate. As a result, the charge transfer resistance was high, and the corrosion rate was the lowest, indicating the mitigation of pitting corrosion.

Under actual service conditions, the sodium lactate contents of 0 mg/L (no carbon source was provided) and 3500 mg/L (carbon source was extremely abundant) were both extreme conditions. It was common that the production water was mixed with some organic substances to provide partial organic carbon source, which corresponded to the three groups of tests with a sodium lactate content ranging from 350 to 1750 mg/L. Under this circumstance, there may be a proper range of carbon source content, which could not only ensure the rapid reproduction of bacteria in the early stage to form a complete biofilm, but also create a “starving” environment for bacteria to take metals as the electron donors. In this case, the charge transfer resistance was reduced, and the corrosion rate was increased, indicating the aggravation of pitting corrosion.

In particular, it should be pointed out that the content of planktonic bacteria was generally several orders of magnitude lower than that of bacteria attached to the substrate surface. Hence, the content of planktonic bacteria measured in the field could only be used as a reference for the growth state of bacteria. For example, the content of planktonic bacteria at the sodium lactate content of 700, 1750, or 3500 mg/L was higher than that at the sodium lactate content of 350 mg/L, whereas the corrosion rate and pitting depth were significantly lower than those when the sodium lactate content was 350 mg/L. Of course, the overall corrosion and the evolution of pitting were related to various factors such as the growth of bacteria, the formation and damage of film layers, the temperature, the CO_2_ partial pressure, and the replenishment of organic carbon sources, and the impacts of these factors need to be further studied.

## 5. Conclusions

(1) Carbon sources serve as organic electron donors to SRB, affecting the source of electrons needed for bacterial metabolism. Reduction in the carbon source content could cause the bacteria to “starve” for electrons directly taken from the metal, increasing the MIC. The corrosion rate reached the maximum value of 0.077 mm/a at a sodium lactate level of 350 mg/L. With the increase in sodium lactate content, the corrosion rate gradually decreased to 0.022 mm/year.
(2) The low carbon source content resulted in a reduction in the number of SRB cells, but the corrosion rate and pitting depth were higher. Carbon starvation increased the EET activity of SRB and accelerated the SRB-MIC of carbon steel. The planktonic bacterial content measured during routine operation and maintenance should not be used as an indicator to determine the degree of SRB-MIC, while the sessile cell count is the main point of assessment.
(3) In future work, we will further investigate the impact of time and other organic carbon sources.

## Figures and Tables

**Table 1:** Composition of production water.

Constituent	NaCl	CaCl_2_	MgCl_2_·6H_2_O	Na_2_SO_4_	NaHCO_3_
Mass/mg·L^−1^	32,654	3301	926	130	225

**Table 2:** Composition of culture medium.

Constituent	K_2_HPO_4_·3H_2_O	NH_4_Cl	Na_2_SO_4_	CaCl_2_	MgSO_4_	Sodium Lactate	Yeast Water	Fe(NH_4_)_2_•(SO_4_)_2_	Vitamin C
Mass/mg·L^−1^	500	1000	500	100	2000	3500	1000	300	100

**Table 3:** Chemical composition of L245 carbon steel (mass fraction) %.

Element	C	Si	Mn	P	S	Cr	Ni	Mo	V	Nb	Ti	Fe
Content	0.203	0.255	0.397	0.0189	0.0139	0.023	0.021	0.019	0.0015	0.003	0.0025	Bal.

**Table 4:** Experimental phenomenon.

Sodium Lactate Content/mg·L^−1^	Odor	Solution Color	Sediment Color
Control	None	Faint yellow	None
0	Slight odor	Faint yellow	None
100	Slight odor	Faint yellow	None
200	Slight odor	Faint yellow	Faint yellow
350	Slight odor	Faint yellow	Faint yellow
500	Slight odor	Faint yellow	Faint yellow
700	Rotten egg odor	Yellow	Yellow
1750	Strong rotten egg odor	Bright yellow	Black
3500	Strong rotten egg odor	Black	Black

**Table 5:** EDS spectrum analysis results derived from the sample surfaces (wt.%).

Sodium Lactate Content/mg·L^−1^	Control	0	350	700	1750	3500
C	0	10.29	9.82	8.5	22.45	5.61
O	3.53	8.21	15.35	40.55	41.52	32.99
S	0	0.46	12.01	1.83	1.98	4.35
Ca	0	0.96	3.05	12.7	22.56	3.37
Fe	96.47	80.08	59.77	36.42	11.49	53.68
Total	100	100	100	100	100	100

**Table 6:** EIS parameters.

Sodium Lactate Content/mg·L^−1^	*R*_s_/Ω·cm^2^	*Q*_bc_/ Ω^−1^·cm^−2^·S^n^	*R*_b_/Ω·cm^2^	*Q*_dl_/ Ω^−1^·cm^−2^·S^n^	*R*_ct_/Ω·cm^2^	*R*_bc_ + *R*_ct_/Ω·cm^2^
Control	14.23	1.239 × 10^−3^	3312	5.391 × 10^−4^	7060	10,372
0	19.06	1.89 × 10^−3^	341.3	1.74 × 10^−3^	5425	5766.3
350	3.23	4.502 × 10^−3^	33.9	1.891 × 10^−3^	1279	1312.9
700	17.11	1.938 × 10^−3^	20.3	5.981 × 10^−3^	7052	7072.3
1750	20.89	1.079 × 10^−3^	12.9	2.242 × 10^−3^	8626	8638.9
3500	18.21	9.829 × 10^−4^	21.1	1.712 × 10^−3^	9620	9641.1

**Table 7:** Comparison of the MIC patterns of metallic substrates by bacterial biofilm in various culture media within this study and those reported in the literature.

S/No	Metal Substrate	Type of Bacterium/Culture Duration	Modification of Test Media	Impact of Organic Carbon Source Reduction on Steel Corrosion	[Ref.]
1.	L245 steel	*desulphovibrio/*10 days	Production water with different contents of sodium lactate	In the experiments with sodium lactate content ranging from 0–3500 ppm, the corrosion rate and pitting depth were the highest at 350 ppm	This study
2.	C1018 carbon steel	Desulfovibrio vulgaris/7 days	No modification; only reduction in lactate and citrate content	Weight loss increased, and larger pit width was observed on steel in 90% and 99% CSR medium. Deeper pits were observed in 99% CSR compared with 90% CSR.	[12]
3.	C1018 carbon steel	*D. Vulgaris/*3 days in SRB medium and 7-day starvation experiment	Modified ATCC 1249 media by reducing lactate and citrate amounts at different levels	A 20% carbon source led to the highest weight loss, while 100% had the lowest. Corrosion by SRB is due to electron harvest from extracellular iron oxidation by SRB, which belongs to extracellular electron transfer MIC (EET-MIC)	[13]
4.	Pipeline (X70) steel	*Desulfovibrio desulfuricans/*30 days	With simulated CO_2_-saturated oilfield produced water	Severe anodic steel dissolution is observed at the end of the culture period within the test media due to SRB-led MIC and CO_2_ corrosion.	[19]
5.	Pipeline (X80) steel	Desulfotomaculum nigrificans and Pseudomonas sp./21 days	With simulated CO_2_-saturated oilfield produced water	Corrosion increased with length of incubation time of steel substrate within the culture medium; corrosion is attributed to MIC and CO_2_ corrosion.	[20]
6.	C1018 carbon steel	Pseudomonas aeruginosa/7 days	No modification; only reduction in carbon source	Steel substrates in 90% and 100% CSR medium had higher mass losses and wider pit depths compared with the control.	[21]
7.	C1018 carbon steel	Thermophilic *Archaeoglobus fulgidus* strain/7 days	No modification; only reduction in carbon source	The 7-day incubation period under carbon starvation increased sessile cell counts and also increased the thickness of *Archaeoglobus fulgidus* biofilms, leading to corrosion and deeper pit depths.	[22]

## Data Availability

Data are contained within the article.

## Nomenclature

SRB	Sulfate-reducing bacteria
TGB	saprophytic bacteria
IOB	iron-oxidizing bacteria
MIC	microbiologically influenced corrosion
M-MIC	metabolite MIC
EET-MIC	extracellular electron-transfer MIC
MPN	most probable number
SEM	scanning electron microscopy
SE	secondary electron
EDS	energy-dispersive spectrometry
EIS	electrochemical impedance spectroscopy
SCE	saturated calomel electrode
RE	reference electrode
CE	counter electrode
WE	working electrode
PTFE	polytetrafluoroethylene
